# A reconciling vision of the Adriatic-Ionian Bimodal Oscillating System

**DOI:** 10.1038/s41598-023-29162-2

**Published:** 2023-02-09

**Authors:** Gian Luca Eusebi Borzelli, Sandro Carniel

**Affiliations:** 1 Center for Remote Sensing of the Earth (CERSE), Via dei Vascellari 40, 00153 Rome, Italy; 2 NATO STO-CMRE, Centre for Maritime Research and Experimentation, La Spezia, Italy

**Keywords:** Ocean sciences, Physical oceanography

## Abstract

The bimodal oscillating system (BiOS) consists in an oscillation of the Ionian Sea surface structure with period of 12–13 years, which reflects in a near-surface circulation inversion. BiOS regimes are deeply interconnected with the circulation patterns of the Eastern Mediterranean, and it is a dominant process governing water masses formation, air-sea fluxes and bio-geochemical properties, which impacts living organisms. The BiOS has been partially explained as a self-sustained oscillation maintained by the interplay between Adriatic dense water formation and changes in the relative volume of waters of Levantine and Atlantic origin entering the Adriatic; however, attempts have also been made to explain the BiOS in terms of atmospheric-related processes. Despite the intensive research aiming at reproducing this oscillating system, the fundamental question “which is the source of energy necessary to initiate the BiOS?” has, until now, remained unanswered. The scope of this paper is two-fold. First, we document that, since 1993, two periods in the BiOS can be observed: a first one, between 1993 and 2017, during which the BiOS damped up to nearly disappear, with e-folding time of 11 years; and a second one, starting in 2017, during which the BiOS revitalized. Then, we propose here an analytical model that, under a two-layer ocean assumption, shows how it is possible for winds rotating in the same direction to initiate oscillations of the free surface, as result of the competing effects of wind and internal fluid pressure fields. The proposed model forced with wind data could successfully reproduce the characteristic time scales of the BiOS cycle over the period 1993–2019, and is therefore offered as a novel vision explaining the originating mechanism as the basis of its initiation, as well as a fundamental tool to address possible BiOS regimes in future climate scenarios.

## Introduction

Thermohaline oscillations of the Adriatic-Ionian system (see e.g.^1^) have been studied extensively since Buljan^2^ noticed a decadal variability in the Adriatic salinity. However, despite these efforts, the reconstruction of the dynamics underlying this variability remained a puzzle until 2010, when Gačić et al.^3^ related changes in the hydrographic properties of the Adriatic water masses to decadal oscillations of the Northern Ionian Gyre (NIG)*.* They noticed that there are two basic circulation regimes in the Northern Ionian (NI): cyclonic and anticyclonic. Depending on their relative prevalence, different volumes of waters of Levantine and Atlantic origin enter the Adriatic, changing and triggering the decadal variability of the thermohaline properties of the whole Adriatic. This mechanism, named Adriatic-Ionian Bimodal Oscillating System (BiOS), correlates the Adriatic oceanographic variability to changes in the NI dynamics^4^. Besides hydrology, the BiOS influences the biodiversity of the Adriatic and Levantine sea^5,6^. It has been shown, for instance, that it modulates the connectivity patterns between different Mediterranean ecosystems favoring, in its cyclonic mode, Lessepsian migrations in the Adriatic^7^, which modify fish stocks in this region^8^. Understanding the mechanisms underlying the BiOS is therefore essential to forecast its cycles in relation to their social and economical impacts.

There are two generally accepted theories to explain the physics underlying the oscillation of the NI near-surface dynamics. The first theory relates NI current reversals to changes in the wind stress curl^9–11^; the second relates current reversals of the Ionian near-surface circulation to baroclinic (internal) vorticity production, induced by changes in the horizontal pressure gradient due to injections of Adriatic Deep Water (AdDW)^12^. Rubino et al.^13^ and Gačić et al.^14^, relying on tank experiments and numerical modeling, demonstrated that the polarity switch of the near-surface circulation in the NIG can be indeed induced by the injection of dense water on a sloping bottom.

The two theories offer an explanation about the physics underlying the BiOS paradigm, but both have flaws. Specifically, the first theory, although based on the most natural approach to explain the inversion of the near-surface Ionian current, is not supported by data published in the available literature^3,12,15^, which show that variations in the oceanic near-surface vorticity are, on average over the NI, decorrelated from the wind vorticity. When tackled using the second theory, the BiOS is regarded as a self-sustained, quasi-perennial, oscillation of the NI sea surface structure; however, the question on the sources of energy and momentum necessary to initiate and sustain the oscillation, is not addressed. Moreover, according to this explanation^3,12^, the BiOS paradigm requires that changes in the sea surface structure of the NI are to be driven solely by the deformations of the interface between deep and intermediate waters, hence implicitly assuming that the interface between surface and intermediate waters has no role in the transition between the two states of the Ionian dynamics. More specifically, at first approximation, the vertical structure of the NI is characterized by three water masses: a surface layer of waters of Atlantic origin (from the surface to approximately 150 m), an interface layer of waters of Levantine origin (between 150 and 500 m) and a deep layer of waters of Adriatic and/or Aegean origin (from 500 m down to the bottom). To drive the mechanism underlying the transition of the Ionian dynamics, according to the second theory, there is therefore only the interface between the deep and Levantine waters, the basic assumption being that the surface and intermediate layers, during the transition, move rigidly in phase. This assumption is indirectly confirmed by the fact that changes in the hydrographic properties of the Adriatic and the Eastern Mediterranean occur in phase with the Ionian dynamics^3,4,16–19^ and supports the hypothesis that the Ionian circulation can be described reasonably well by a double-layered system. Therefore, in the following, consistently with the second theory explaining the NI oscillations, we shall describe the vertical structure of the Ionian Sea as a two-layer system, drawing however the attention on this dynamic peculiarity, which would require research aiming at clarifying how the different baroclinic components interact with each other in order to determine the horizontal circulation patterns.

In order to assess the role of winds as the dominant source determining changes in oceanic near-surface vorticity, we compared the wind vorticity (inferred from 6 h ERA-Interim winds in the period 1 January 1993–19 August 2019 averaged over the NI) with the mean variation of the oceanic near-surface vorticity over the same area (inferred from the daily Absolute Dynamic Topography (ADT) deduced from Sea Surface Height (SSH) data in the period 1 January 1993–31 December 2019). The resulting degree of correlation between the wind stress vorticity and the variation in the oceanic near-surface vorticity is low, confirming that the wind vorticity is not the main actor here. Moreover, performing an Empirical Orthogonal Function (EOF) analysis of the SSH time series, we show that in the period 1993–2017 the BiOS can be described as a damped oscillation with damping time of 11 years and a period of approximately 12–13 years.

In order to explain BiOS damping and revitalization, we propose here a simple analytical theory, which assumes that the Ionian is a double-layered system. The model shows that winds rotating constantly in the same direction can initiate oscillations of the free surface and, as a consequence, of the interface between the layers, as the result of the competing effects of the wind and the internal fluid pressure. On the basis of available wind data and reasonable assumptions on the stratification of the water column, we show that this theory provides estimates of the BiOS characteristic time scales compatible with the observations.

## Results

**Figure 1:**
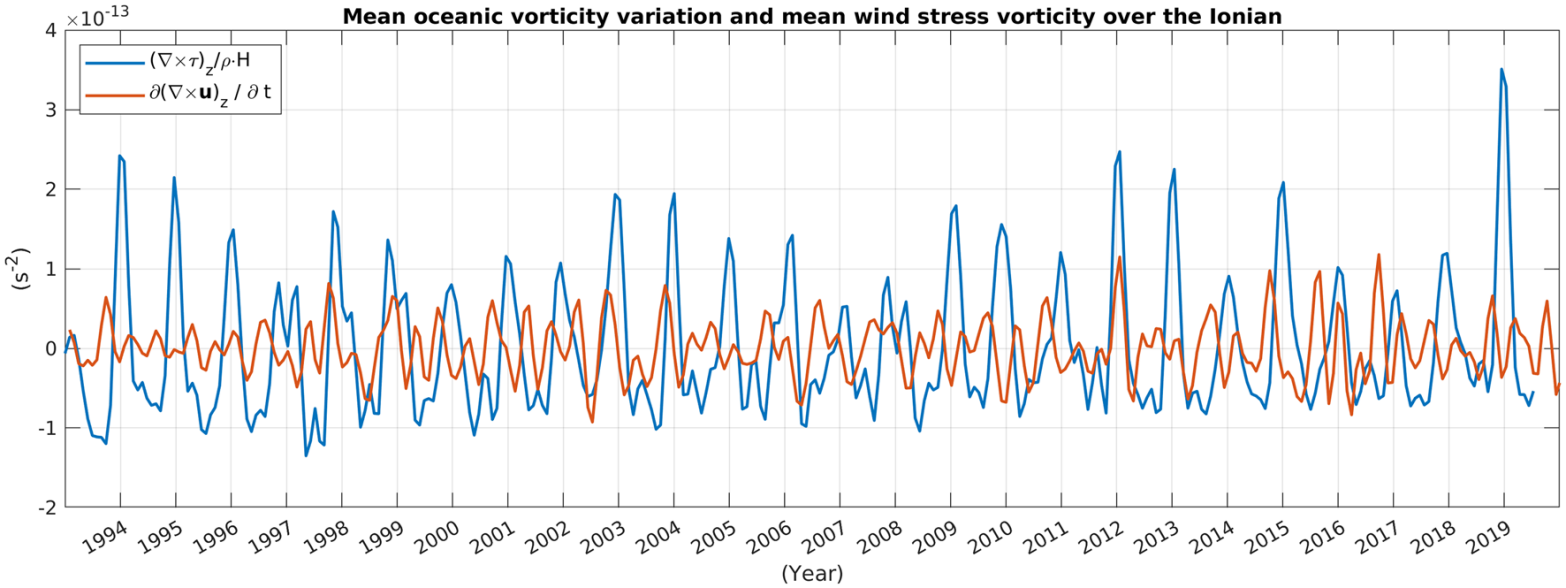
Low-passed (cutoff frequency 1/120 days^-1^) vorticity source due to wind stress in the period 1 Jan, 1993–19 Aug, 2019 (blue line) and variation in the oceanic relative vorticity in the period 1 Jan, 1993–31 Dec, 2019 (red line), averaged over the NI (with reference to Fig. S4, the “Eddy Region”). Correlation between red and blue line: − 0.021. This figure has been created using MATLAB software package version 2018a (www.mathworks.com).

In Fig. 1 we present the average vorticity over the NI (with reference to Fig. S4, the region indicated as “Eddy Region”-ER), computed from wind stress data obtained from ERA-Interim, 6-Hourly winds in the period 1 Jan 1993–19 Aug 2019, along with the variation of the oceanic relative vorticity, averaged over the same region and computed using SSH data (i.e. *ζ* = [*curl*(***u***)]_*z*_ = − (*g*/*f*)∇^2^*ssh*, in the period 1 Jan 1993–31 Dec 2019). The degree of correlation between these data sets is quite small (− 0.021), indicating that the wind cannot be considered the dominant source of vorticity determining changes in the oceanic relative vorticity.

**Figure 2:**
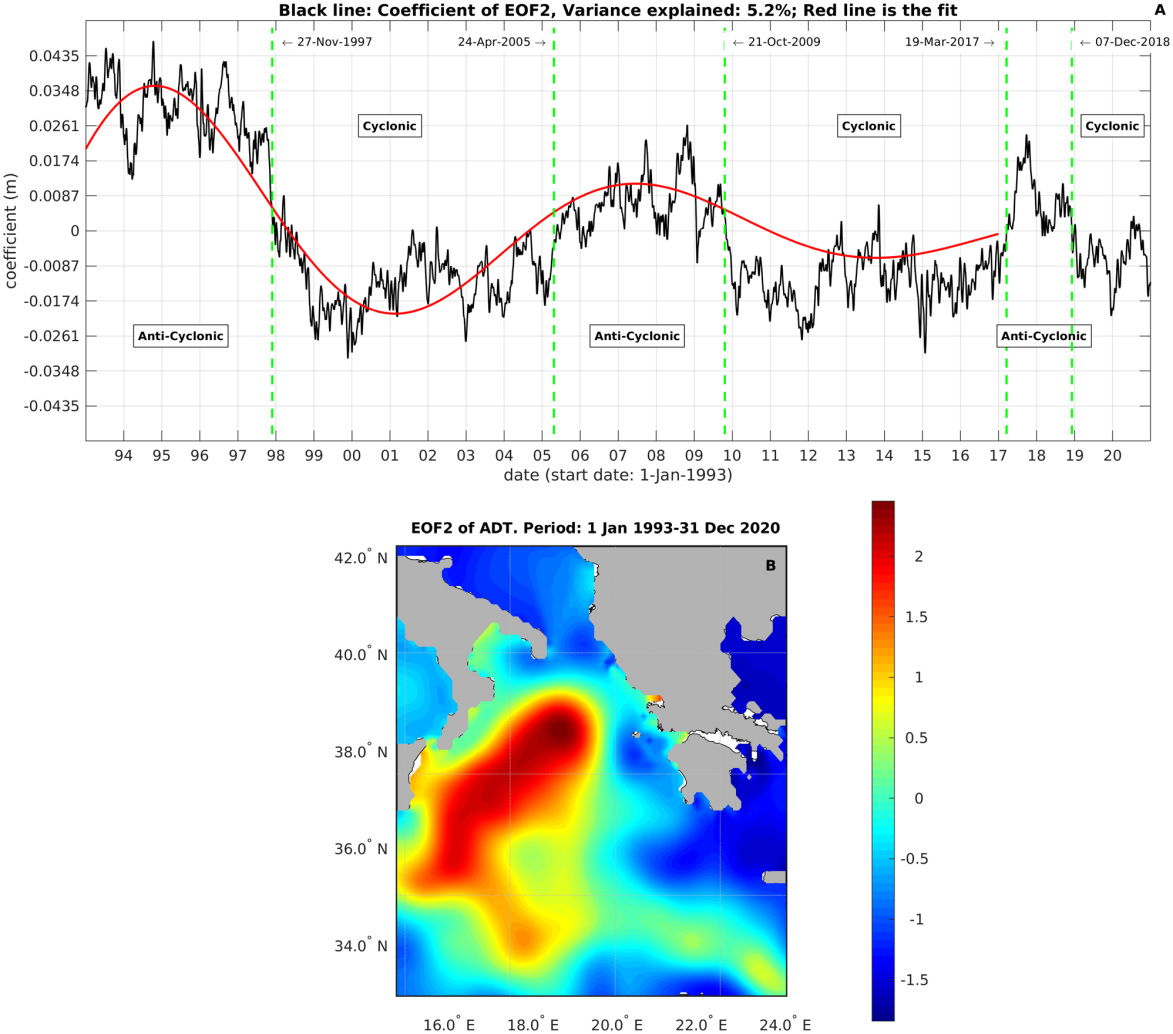
Empirical Orthogonal Function analysis of SSH. (**A**) the black line represents the coefficient of EOF2, the red line its fit to the function *e*^−(*t*/*τ*)^cos(*2π*·*t*/*T* + *φ*); fitted values are: *T* = 12.6 years, *τ* = 11.3 years. (**B**) the second EOF mode. This figure has been created using MATLAB software package version 2018a (www.mathworks.com).

**Figure 3:**
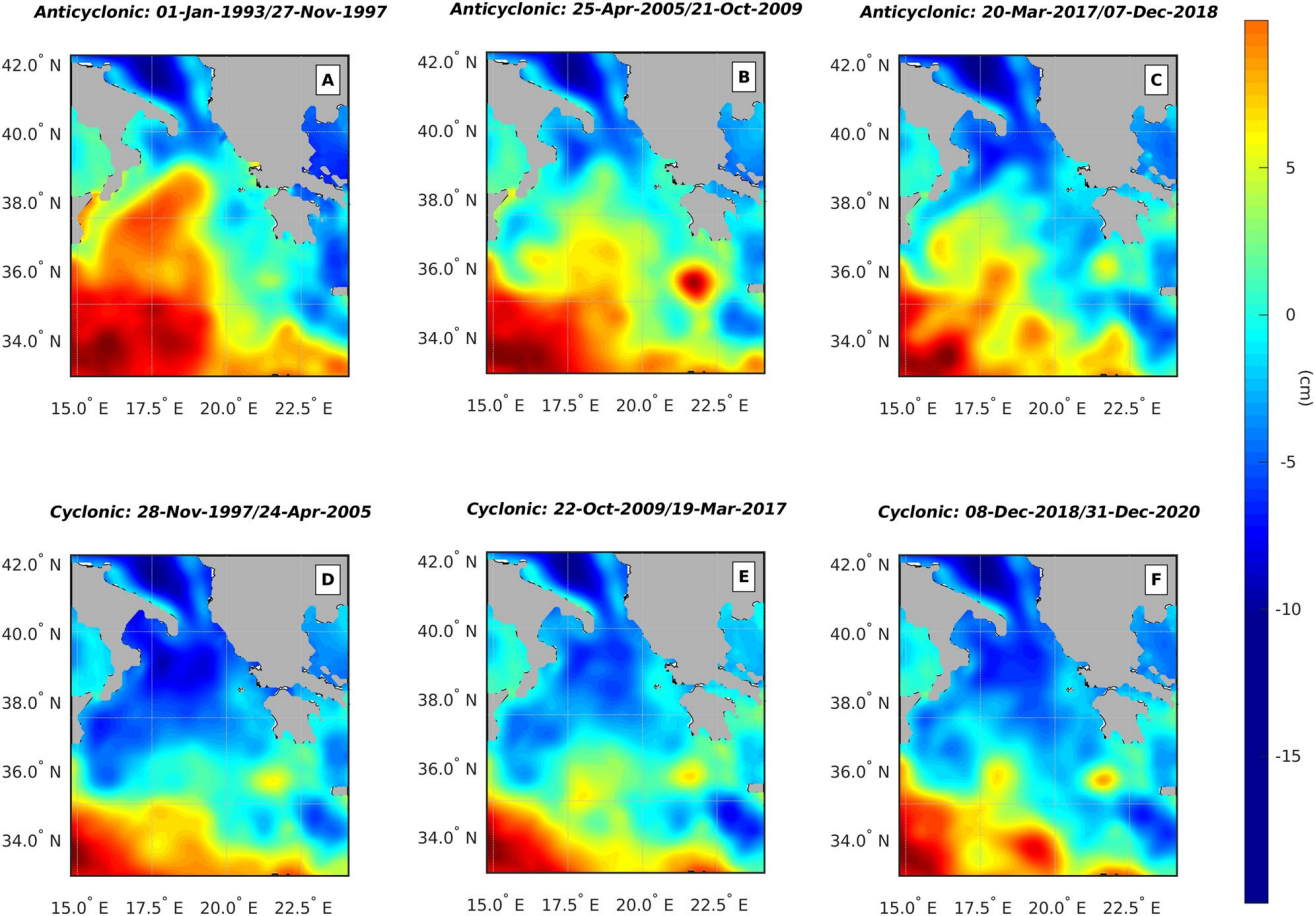
Average SSH over the anticyclonic (**A-C**) and cyclonic periods (**D-F**). This figure has been created using MATLAB software package version 2018a (www.mathworks.com).

In “Material and methods” Section, we have proved that at least 87% of the NIG variability is explained by the second EOF; in Fig. 2, the second spatial mode of the EOF analysis (Fig. 2B) of the ADT time series and its temporal coefficient (Fig. 2A) are depicted and show that the inversion of the Ionian current can be described as a damped oscillation of the ADT, with period of 12–13 years and damping time of 11 years. In Fig. 3, to provide a synoptic view of the BiOS evolution and document its damping, the average ADTs over the different cyclonic and anticyclonic periods are shown.

The model developed in the supplementary material and used to describe the NIG oscillation is an oscillatory model forced by the wind. In brief (for the definition of symbols see “Material and methods” Section), according to this model, the wind sustains a deformation of the free surface. When the wind stops blowing, the system returns to its state of equilibrium through damped oscillations propagating within the fluid with velocity *c*, characteristic length scale *L* and oscillations decay over a time scale of *τ* = *L/c*. In order to verify this model we have to assume some values for the stratification necessary to evaluate the parameters in Eqs. (2) of the “Material and methods” Section. In the investigated region (see Fig. S4 for reference), reasonable values of the parameters quoted in Eq. (2) are *H*_1_ ≈ 500 m, *H*_2_ ≈ 1700 m,* ρ*_1_≈ 1028.6 kg/m^3^, *ρ*_2_ ≈ 1029.2 kg/m^3^ (see Supplementary Material, Fig. S1, for the definition of symbols)*,* which provide an internal wave velocity *c* ≈ 1.49 m/s. Furthermore, note that in the definition of *ω*_*m*_ (Eq. 2 of the “Material and methods” Section), *θ*_0_ is expressed in units (damping length)/(radius of the gyre); this implies that the oscillation frequency is independent of the size of the gyre (and is expressed in cycles/s and not in rads/s). With these positions and the definitions of damping length (*L*), damping time (i.e. *τ* = (damping length)/*c* = *L*/*c*) and oscillation frequencies (*ω*_*m*_), derived in the Supplementary Material and quoted in the “Material and methods” Section (Eq. 2), we get the results presented in Table 1, which summarizes the values of these parameters in relation to different values of the tangential component of the wind stress. The first line of Table 1 shows the values of the damping length, damping time and oscillation periods of the first three oscillatory modes of the sea surface obtained from the model Eqs. (2), with *τ*_*θ*_ = 4.6·10^–3^ N/m^2^ (wind intensity of ~ 1.83 m/s), which is the value of the tangential wind stress averaged in the period 1 Jan 1993–31 Dec 2016, over the region denoted as “Eddy Region” (ER) in Fig. S4. The second line of Table 1 shows the same parameters obtained though the model equations, with *τ*_*θ*_ = 10.1·10^–3^ N/m^2^ (wind intensity of ~ 2.65 m/s), which is the value of the tangential wind stress averaged in the period 1 Jan, 1993–31 Dec, 2016, over the entire study region, which comprises part of the Cretan Sea, a portion of the Southern Adriatic and a slight piece of the Tyrrhenian. Line 3 of Table 1 shows damping length, damping time and oscillation periods of the first three oscillatory modes of the sea surface obtained setting *τ*_*θ*_ = 7.2·10^–3^ N/m^2^, (wind intensity of ~ 2.23 m/s), which is the value of the tangential wind stress averaged in the period 1 Jan, 2017–31 Dec, 2018 (wind data are available until 19 Aug, 2019; so, in the wind averaging process, wind data between 1 Jan, 2019 and 31 Aug, 2019 have been disregarded to avoid undesirable seasonal effects), over the ER. Comparing line 1 and 3 of Table 1, it is evident that, according to the model equations, owing to a slight increase in the tangential wind component during 2017–2018 with respect to 1993–2016, the oscillation period of the first sea surface oscillatory mode drops from 12.98 years (line 1 of Table 1) to 8.3 years (line 3 of Table 1) and the damping time decreases from 13.61 to 8.74 years.

**Table 1:** Variation of damping length, damping time and oscillation period of the first three sea surface oscillation modes obtained with different values of *τ*_*θ*_.

	Damping length (L)	Damping time (L/c)	Oscillation period (m = 1)	Oscillation period (m = 2)	Oscillation period (m = 3)
*τ*_*θ*_ = 0.0046 N/m^2^ (*Mean Over ER*); *θ*_*0*_ = 10 ∙ L/r	6.39∙10^8^ m	13.61 years	12.98 years	11.52 years	9.9 years
*τ*_*θ*_ = 0.0101 N/m^2^ (*Mean Over SR*); *θ*_0_ = 10 ∙ L/r	2.9∙10^8^ m	6.2 years	5.91 years	5.25 years	4.5 years
*τ*_*θ*_ = 0.0072 N/m^2^ (*Mean Over ER*); *θ*_*0*_ = 10 L/r	4.1∙10^8^ m	8.7 years	8.34 years	7.4 years	6.36 years
*u* = 2.0 m/s; *τ*_*θ*_ = 0.0058 N/m^2^; *θ*_*0*_ = 10 ∙ L/r	5.1∙10^8^ m	10.9 years	10.37 years	9.2 years	7.91 years
*u* = 2.5 m/s; *τ*_*θ*_ = 0.0090 N/m^2^; *θ*_*0*_ = 10 ∙ L/r	3.26∙10^8^ m	6.95 years	6.63 years	5.89 years	5.06 years
*u* = 3.0 m/s; *τ*_*θ*_ = 0.0130 N/m^2^; *θ*_*0*_ = 10 ∙ L/r	2.27∙10^8^ m	4.83 years	4.61 years	4.09 years	3.51 years

Wind stresses values in line 1 and 2 are obtained averaging the tangential wind stress in the period 1 Jan, 1993–31 Dec, 2016, over the “Eddy Region” (ER) and over the “Study Region” (SR) (Fig. S4), respectively. The value of *τ*_*θ*_ in line 3 is obtained by averaging the tangential wind stress over the “Eddy Region” (ER) in the period 1 Jan, 2017–31 Dec, 2018. Lines 4–6 are meant to provide the reader with the order of magnitude of the variations of oscillatory parameters of the NIG in relation to wind intensity.

Lines from 4 to 6 in Table 1 are meant to provide the reader with the order magnitude of the changes in the characteristic parameters of the sea surface oscillations in relation to changes in the tangential wind stress.

In all the illustrated cases, the damping time is comprised between 4.8 and 13.6 years and, remarkably, characteristic parameters of the sea surface oscillation, obtained with the tangential wind stress averaged over the ER in the period 1993–2016, are coherent with observed values of the damping time and oscillation period (see Fig. 2A).

## Discussion

The Adriatic-Ionian BiOS is commonly described as a quasi-periodic oscillation between two circulation regimes, cyclonic and anti-cyclonic, of the NIG. It has been widely demonstrated that the oceanography of the entire Eastern Mediterranean is remarkably influenced by the BiOS phases^16^ and, probably, also the Eastern Mediterranean Transient^20^ can be associated with the anticyclonic mode of the NI circulation^12^. According to this interpretation, the NI acts as a distributor of water masses with different thermohaline properties in the adjacent basins: the Ionian cyclonic or anticyclonic circulation determines the alternate advection into the Adriatic or into the Cretan Sea of saltier water from the Levantine basin, or fresher water of Atlantic origin, and therefore modifies the thermohaline properties and circulation of the Adriatic and Levantine basins^3,16–19^.

There are two theories to explain the physics underlying the NI near-surface dynamics reversal. The first one relates NI current reversals to changes in the vertical component of the wind stress curl^9–11^. The second one relates current reversals of the Ionian near-surface circulation to baroclinic (internal) vorticity production, induced by changes in the horizontal pressure gradient due to injections of AdDW^3,12^. More specifically, based on the fact that internal processes can outweigh wind stress in near-surface oceanic vorticity production^12^, the NIG inversion has been traditionally explained as an oceanic feedback, in which the redistribution of water masses in the depths of the Ionian, determined by changes in the basin circulation, induces variations in the horizontal pressure gradients and, in this way, sustains the NIG inversion.

By using a high-resolution wind data set and confirming the observations of other authors^3,12,15^, we have shown that the spatial average vorticity source associated with the wind stress curl is not able to sustain inversions of the Ionian near-surface current (see Fig. 1). Although the spatial averaging process could filter out localized wind patterns and hide localized vorticity sources due to the wind stress, data presented here, along with those published in the available literature^3,12,15^, show that the inversions of the Ionian circulation can not be explained purely in terms of wind stress vorticity. However, the second mechanism, generally forwarded to explain the NI dynamics, also appears to have its flaws. Indeed, here we have shown (Figs. 2 and 3) that the BiOS is a damped oscillation with damping time of, approximately, 11 years, which is an observation inconsistent with the view of a self-sustained, quasi-perennial oscillation. Furthermore, Gačić et al.^21^ observed a premature inversion of the Ionian current from cyclonic to anti-cyclonic in relation to the massive formation of dense water in the Adriatic during the winter of 2012. Here we have documented that this transition did not actually take place (Figs. 2 and 3) and, net of damping, the Ionian circulation remained cyclonic until spring 2017, opening a problem in interpreting the role of the Adriatic dense water in the NIG inversion.

In order to explain these inconsistencies, we have developed an analytical internal Kelvin-like wave model. This model is inspired by the fact that the sub-surface memory of the ocean (i.e. the energy stored in the water column) depends on the shape of the isopycnal surfaces, which are systematically deformed by the action of a rotating wind. The fluid is initially at the hydrostatic equilibrium and the equilibrium is maintained by the perfect Svedrup balance between the internal fluid pressure, which determines the progressive deformation of the isopycnal surfaces, and the wind. When the internal fluid pressure exceeds the action of the wind (parameterized in the Supplementary Material as a “Small Perturbation to the Equilibrium State”), the equilibrium between the ocean and the external forcing is broken, the potential energy stored in the water column is made available and the system begins to oscillate. To comfort ourselves and the reader that the data are consistent with the Kelvin-like wave model, we showed that the second EOF mode describes the NIG variability (see “Material and methods” paragraph and Supplementary Material, Fig. S3) and have performed a fit between the temporal amplitude of the second EOF and the model equations (Eq. 1 of the “Material and methods” Section) to obtain fitting parameters consistent with observations of the NIG variability. Furthermore, by using averaged values of measured tangential wind stresses, using the model Eq. (2) (see the “Material and methods” Section), we reproduced BiOS oscillation frequencies and damping rates consistent with observations. Since the work of Gačić et al.^3^ it was known that the oscillation period of the BiOS was approximately 10 years and a great deal of effort, dealing with numerical modeling and tank experiments (see e.g.^13,14^), has been spent in order to reproduce this frequency, however quite unsatisfactory. Here we have shown, for the first time, that the observed frequencies of the BiOS oscillation are consistent, though the Kelvin-like wave model equations, with the observed winds (Table 1). This remarkable result is further confirmed by the fact that we have identified two periods in the BiOS: one spanning 1993–2016, characterized by an oscillation period of 12–13 years and damping time of 11 years, and another one spanning the period 2017–2020. This period is too short to perform a credible fitting, but a visual inspection of Fig. 2A suggests that, in the second period, both the BiOS oscillation period and damping time decreased. This may happen primarily because of an intensification of the tangential wind component and, indeed, average values of the tangential wind component over the NIG region in the period 2017–2018 do increase. According to the Kelvin-like model, this increase in the tangential wind stress should produce a decrease in the BiOS oscillation period from 12.98 to 8.3 years, and a decrease in the damping time from 13.61 to 8.74 years (compare line 1 and 3 of Table 1). If this slight increase in the tangential wind intensity will continue, we can therefore expect that the BiOS oscillation will be faster in the next years.

## Conclusions

In this research we investigated the inversions of the Northern Ionian (NI) near-surface current in the period 1993–2020, proposing an alternative analytical model to explain its recurrent inversion. The model relies upon the concept that even winds constantly rotating in the same direction can provide the energy and momentum necessary to achieve oscillations in the Sea Surface Height. The model has been developed from a two-layer approximation, a common assumption used by other BiOS theories, justified by the fact that oscillations in the thermohaline properties of basins adjacent to the Ionian are in phase with the BiOS rhythm. During the cyclonic phase we have also seen that even an exceptional production of dense water in the Adriatic, although leaving its signature on the intensity of the cyclonic near-surface circulation, is not per se capable of sustaining a transition from cyclonic to anti-cyclonic, as would be prescribed by canonical BiOS theories. Using the model proposed here, on the basis of reasonable assumptions on the stratification of the Ionian Sea and daily wind data, we obtained estimates of the characteristic time-scales of the BiOS consistent with observations. Additional research aiming at reproducing accurately the BiOS cycles from 1979, date from which the ERA-Interim wind data are available, are planned. Furthermore, we consider as necessary further research on how the interactions between different baroclinic modes determine the horizontal circulation patterns during the Northern Ionian Gyre transition and on separating the different contributions to the Northern Ionian Gyre transition of the Eastern Mediterranean Transient, the mechanism described here and the redistribution of water masses in the Ionian abyss. Last but not least, we believe this contribution may offer valuable insights on assessing possible future shifts of BiOS regimes in the climate scenarios.

## Materials and methods

In our representation the ocean is described as a double-layered system in polar coordinates, with origin at the center of the NIG, forced by a wind stress with components (*τ*_*r*_, *τ*_*θ*_) (for details on the model equations and derivations of different formulas see Supplementary Material). Seeking solutions with radial velocity equal to zero, one gets that the surface layer obeys to the following equations

1 $$\begin{gathered} \sigma \left( {\theta ,r,t} \right) = \sum\limits_{m = 1}^{\infty } {e^{{ - \left( \frac{r}{L} \right) \cdot \theta }} \cdot \sin \left( {k_{m} \cdot r \cdot \theta } \right) \cdot \left[ {a_{m} \left( r \right) \cdot \cos \left( {\omega_{m} t} \right) + b_{m} \left( r \right) \cdot \sin \left( {\omega_{m} t} \right)} \right]} \\ = \sum\limits_{m = 1}^{\infty } {e^{{ - \left( \frac{r}{L} \right) \cdot \theta }} \cdot \sin \left( {k_{m} \cdot r \cdot \theta } \right) \cdot A_{m} \left( r \right) \cdot \cos \left( {\omega_{m} t + \varphi_{m} } \right)} \\ \end{gathered}$$

where in the last step, *A*_*m*_∙cos*Ф*_*m*_ = *a*_*m*_, *A*_*m*_∙sin*Ф*_*m*_ = − *b*_*m*_, we assumed that the motion is constrained along the gyre (i.e. *θ* = *ct*/*r*) and we have set

2 $$\gamma = \frac{{\tau_{\theta } H_{2} }}{{\rho_{1} H \cdot H_{1} }};\;L = \frac{{2 \cdot c^{2} }}{\gamma };\;k_{m} = \frac{m\pi }{{r\theta_{0} }};\;\omega_{m}^{2} = \left( {\frac{\gamma }{2 \cdot c}} \right)^{2} + m^{2} \cdot \left( {\frac{c\pi }{{r\theta_{0} }}} \right)$$

with $m = {1} \ldots \infty$, *r* the radius of the gyre, *H*_1_ and *H*_2_ the depth of the surface and bottom layers, *H* = *H*_1_ + *H*_2_ and *c*^2^ = (*g*’*H*_1_*H*_2_/*H*) is the internal wave velocity (all symbols are defined in “Introduction” of the Supplementary Material). In the above equations *θ*_0_ is a boundary condition chosen so as *rθ*_0_ >  > *ρ*.

“6 h" wind data provided by the ERA-Interim project in the period 1 Jan, 1993–19 Aug, 2019 (see https://rda.ucar.edu/datasets/ds627.2/#!description) over the region [33°N− 42°N, 15°E− 24°E] were used to compute the wind stress using the standard formula ***τ*** = *ρ*_*a*_∙*C*_*D*_|***u***_*w*_|***u***_*w*_, where ***u***_*w*_ is the wind velocity, *ρ*_*a*_ the air density (taken as 1.2 kg/m^3^) and *C*_*D*_ is the drag coefficient, taken as 1.2 ∙ 10^–3^ if |***u***_*w*_|< 11 m/s and (0.49 + 0.065|***u***_*w*_|)∙ 10^–3^ if |***u***_*w*_|≥ 11 m/s^22^. Daily mean wind stress values were computed and then transformed to polar coordinates (i.e. (*x*,*y*) → (*r*,*θ*)) using the transformation formula *τ*_*r*_ = *τ*_*x*_cos*θ* + *τ*_*y*_sin*θ*; *τ*_*θ*_ = − *τ*_*x*_sin*θ* + *τ*_*y*_cos*θ*.

Daily SSH data with spatial resolution of 0.125° × 0.125° covering the period 1 Jan, 1993–31 Dec, 2020 were provided by the Copernicus Marine Environment Monitoring Service of the European Union. To analyze the BiOS variability there are two possible approaches, depending on two theoretically equivalent definitions. Indeed, the BiOS may be either defined as an inversion of the near surface geostrophic current (change in the sign of the near-surface relative vorticity) associated with an oscillation of the free surface, or an oscillation of the free surface associated with the reversal of the near surface geostrophic current. So, one possible strategy to analyze the BiOS variability from SSH data relies upon computing the near-surface relative vorticity by estimating the term − (*g*/*f*)^2^*ssh*(***x***,*t*) and then averaging this term over a predefined area, which is assumed to surround the NIG. This is, however, a rather unpractical and quite arbitrary strategy. Unpractical, because performing derivatives on experimental data introduces high frequency noise, which may lead to artifacts; arbitrary, because the averaging area is defined “a priori”, independently of the NIG position, leading, again, to possible errors and miss-interpretations. A much more convenient and reliable approach consists in first isolating the BiOS signal in SSH data, and then analyzing the temporal variability induced in the SSH data set by the BiOS. In 1999 Borzelli and Ligi^23^ demonstrated that EOFs deduced from series of sea surface temperature images multiplied by their temporal amplitude, are particular solutions of the heat diffusion equation with advection. The authors also observed that, if the evolution of a field is described by the linear operator *Ĥ,* which admits separate variable solutions, then the spatial patterns of EOF analysis (*Y*_*n*_(***x***)), multiplied by their temporal amplitude (*c*_*n*_(*t*)), are particular, orthogonal solutions of the equation *Ĥ·φ*(***x***,*t*) = *f*(***x***,*t*), with *f*(***x****,t*) representing an external forcing. Therefore, EOFs have a precise physical meaning and, owing to this, provide a convenient decomposition for isolating the BiOS signal in the SSH time series. Furthermore, the EOF decomposition is convenient because the SSH field is represented as a linear superposition of separate variable modes (i.e. *ssh*(***x***,*t*) = ∑*c*_*m*_(*t*)*Y*_*m*_(***x***)), and the oscillatory model proposed here can be easily tested by fitting *c*_*m*_(*t*) with the model Eq. (1). In order to do this, however, we need to define the EOF, or the group of EOFs, describing the variability in the NIG. In Fig. 2B, the second spatial mode of EOF analysis is shown and, clearly represents an aggregated, loosely elliptical structure, which, multiplied by its temporal coefficient (Fig. 2A), describes an oscillation of the free surface compatible with the BiOS. This is associated with the NIG. Of course, it remains to prove that the only mode of variability associated with the BiOS is indeed associated with the second EOF. To do this, in Fig. S3 of the Supplementary Material, the spatial patterns of EOF1, 3, 4 and 5, which cumulatively explain more than 87% of the SSH data set variance, are shown. Although from the inspection of Fig. S3 interesting structures emerge, none of these can be associated with the BiOS signal, indicating that, at least, 87% of the NIG variability is explained by the second EOF.

To provide quantitative estimates of the BiOS time scales, consistently with the Kelvin-like model Eq. (1), we performed a fit between the second EOF temporal amplitude and a function describing a damped oscillation. This fitting procedure has some important details that need to be reported. According to the BiOS paradigm, the intensity of the Ionian circulation depends on the volume of dense water formed in the Adriatic and/or in the Aegean^13,14^. In the early 90’s, the prevailing source of the Eastern Mediterranean Deep Water (EMDW) was the Aegean Sea, which produced a volume of dense water an order of magnitude larger than the Adriatic one (a phenomenon known as the Eastern Mediterranean Transient, EMT)^20^. Therefore, the observed maximum in the anticyclonic circulation occurring in the early 90’s (Fig. 2A) could be interpreted as a modulation in the BiOS rhythm induced by the EMT, and not as an intrinsic feature of the NIG. However, Incarbona et al.^24^ showed that in 1995 the Adriatic returned to be the predominant source of EMDW; still, performing the fitting in the period 1996–2017, does not appreciably change the fitting parameters (see Supplementary Material, Fig. S2, green line). This result provides a relevant clue that it is not the EMT that determines the maximum in the anticyclonic circulation observed between 1994 and 1996 (Fig. 2A), although strictly speaking it does not provide a definitive proof. Indeed, since reliable information on residence times in the Ionian abyssal waters formed in the Aegean during the EMT period are not available, in principle, at every date subsequent to the beginning of the EMT (i.e. 1988), the Ionian circulation could be influenced by the EMT. Therefore, separating the BiOS intrinsic variations and the EMT contributions in determining the Ionian circulation appears a task that remains open for future research.

## Supplementary Information

Supplementary Information.

## Data Availability

Data used in this research can be downloaded succumbing to European Union (EU) regulations on geophysical data exchange (see https://eur-lex.europa.eu/legal-content/EN/TXT/?uri=celex%3A32014R0377). Original Sea Surface Height (SSH) data are processed and distributed in NetCDF format to registered users by the Copernicus European Marine Service at the following web site: https://resources.marine.copernicus.eu/product-detail/SEALEVEL_EUR_PHY_L4_MY_008_068/INFORMATION after selecting the parameter (i.e. SSH-Absolute Dynamic Topography), the geographic coverage (i.e. 33°N–42°N, 15°E–24°E), the period of interest (i.e. 1 Jan 1993–31 Dec 2019). Original wind data are the horizontal wind components of the ERA-Interim Project, Single Parameter 6-Hourly Surface Analysis and Surface Forecast Time Series, produced by the European Center for Medium Range Weather Forecast (ECMWF) and made available for downloading to registered users in NetCDF format by the National Center for Atmospheric Research (NCAR)—Research Data Archive (RDA, prod. # 627.2) at the following web site: https://rda.ucar.edu/datasets/ds627.2/#!description. ERA-Interim data, before downloading, require the selection of the parameters of interest (i.e. horizontal surface wind components), the geographic coverage (i.e. 33°N–42°N, 15°E–24°E) and the period of interest (i.e. 1 Jan 1993–10 Aug 2019). Daily wind and SSH data over the Ionian Sea, interpolated over a common spatial grid of 0.125° × 0.125° used in this research and software especially developed for this study (https://doi.org/10.6084/m9.figshare.21948512) can be downloaded by users registered to the FigShare.com service at the following web site: https://figshare.com/articles/dataset/Daily_sea_surface_height_wind_stress_and_wind_stress_curl_over_the_Ionian_Sea_in_the_period_1993-2019/21948512. The datasets used and/or analyzed during the current study are also available from the corresponding author on reasonable request.
