# Supplementary material for: A reconciling vision of the Adriatic-Ionian Bimodal Oscillating System
Source: Sci Rep. 2023 Feb 9;13:2334. doi: 10.1038/s41598-023-29162-2 (PMC9911597; doi:10.1038/s41598-023-29162-2)
Supplement: Supplementary file 1 — Supplementary Information. [file 41598_2023_29162_MOESM1_ESM.pdf]

Supplementary Information

1 Internal Kelvin-like Wave forced by a uniformly rotating wind: derivation of model equations

The equation of motion, in vectorized form (in the following, vectors are indicated with arrows and unit vectors in bold), in a two layers ocean are

$$\frac{\partial \vec{v_1}}{\partial t}+\vec{v}_1\cdot\overrightarrow{\nabla_h}\vec{v}_1+2\vec{\Omega}\times\vec{v}_1=-g\overrightarrow{\nabla_h}\eta+\frac{\vec{\tau}}{\rho_1\cdot D_1}$$

$$\frac{\partial \vec{v_2}}{\partial t}+\vec{v}_2\cdot\overrightarrow{\nabla_h}\vec{v}_2+2\vec{\Omega}\times\vec{v}_2=-g\frac{\rho_1}{\rho_2}\overrightarrow{\nabla_h}\eta-g'\overrightarrow{\nabla_h}h$$

$$\frac{\partial D_i}{\partial t}+\overrightarrow{\nabla_h}\cdot(D_i\vec{v}_i)=0$$

where $i=1,2$. The subscript 1 refers to the surface layer and 2 to the bottom. η and h are the surface and interface layer, respectively and ρ_1 and ρ_2 are the densities of the surface and bottom layer (see Figure S1 for details). Note that, from Figure S1, the thickness of the surface and bottom layer can be expressed in terms of deformations of the free surface (η) and interface layer (h) as $D_1=H_1+\eta-h$ and $D_2=H_2+h,$ where H_1 and H_2 are the average thickness of the surface and interface layer.

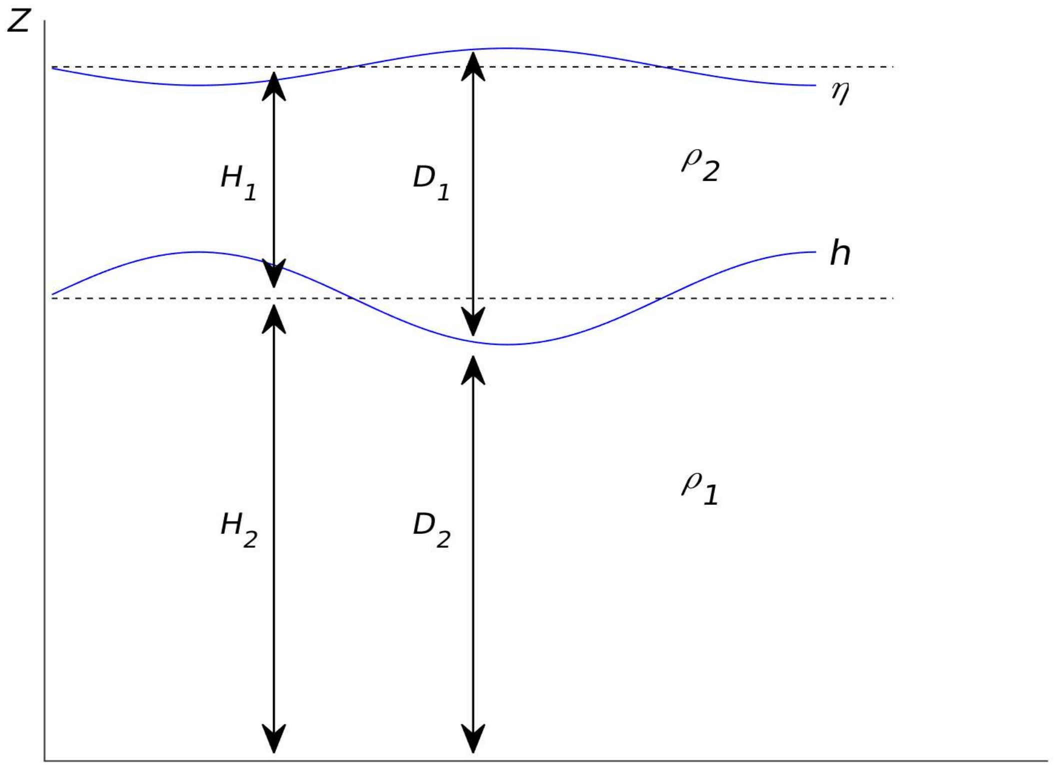

Figure S1. Vertical geometry of the idealized fluid system. This figure has been created using MATLAB software package version 2018a (www.mathworks.com).

Selecting a cylindrical system of coordinates (i.e. $\boldsymbol{x}=\boldsymbol{r}\cdot cos\theta$ - $\boldsymbol{\theta}\cdot sin\theta$; $\boldsymbol{x}=\boldsymbol{r}\cdot sin\theta$ + $\boldsymbol{\theta}\cdot cos\theta$) with center over the center of the eddy, the scalar equations of motion in cyclindrical coordinates are

$$\frac{\partial v_{1r}}{\partial t}+v_{1r}\frac{\partial v_{1r}}{\partial r}+\frac{v_{1\theta}}{r}\frac{\partial v_{1r}}{\partial \theta}-\frac{v_{1\theta}^2}{r}-2\Omega v_{1\theta}\cdot\sin\Lambda=-g\cdot\frac{\partial \eta}{\partial r}+\frac{\tau_r}{\rho_1\cdot D_1} \tag{1a}$$

$$\frac{\partial v_{1\theta}}{\partial t}+v_{1r}\frac{\partial v_{1\theta}}{\partial r}+\frac{v_{1\theta}}{r}\frac{\partial v_{1\theta}}{\partial \theta}+\frac{v_{1r}\cdot v_{1\theta}}{r}+2\Omega v_{1r}\cdot\sin\Lambda=-g\cdot\frac{1}{r}\cdot\frac{\partial \eta}{\partial \theta}+\frac{\tau_\theta}{\rho_1\cdot D_1} \tag{1b}$$

$$\frac{\partial v_{2r}}{\partial t}+v_{2r}\frac{\partial v_{2r}}{\partial r}+\frac{v_{2\theta}}{r}\frac{\partial v_{2r}}{\partial \theta}-\frac{v_{2\theta}^2}{r}-2\Omega v_{2\theta}\cdot\sin\Lambda=-g\cdot\frac{\rho_1}{\rho_2}\cdot\frac{\partial \eta}{\partial r}-g'\cdot\frac{\partial h}{\partial r} \tag{1c}$$

$$\frac{\partial v_{2\theta}}{\partial t}+v_{2r}\frac{\partial v_{2\theta}}{\partial r}+\frac{v_{2\theta}}{r}\frac{\partial v_{2\theta}}{\partial \theta}+\frac{v_{2r}\cdot v_{2\theta}}{r}+2\Omega v_{2r}\cdot\sin\Lambda=-g\cdot\frac{\rho_1}{\rho_2}\cdot\frac{1}{r}\cdot\frac{\partial \eta}{\partial \theta}-g'\cdot\frac{1}{r}\cdot\frac{\partial h}{\partial \theta} \tag{1d}$$

$$\frac{\partial D_i}{\partial t}+\frac{1}{r}\cdot\frac{\partial(r\cdot D_i v_{ir})}{\partial r}+\frac{1}{r}\cdot\frac{\partial(D_i v_{i\theta})}{\partial \theta}=0 \tag{1e}$$

where the wind stress components in cyclindrical coordinates and Cartesian form are related through the relationship $\tau_r = \tau_x cos\theta + \tau_y sin\theta$; $\tau_\theta = -\tau_x sin\theta + \tau_y cos\theta$. To specify the concept of "Subsurface Memory of the Ocean", we consider an initial state at the hydrostatic equilibrium ($v_{i\theta}=v_{ir}=0$), in which the deformation of the free surface (and, therefore, the deformation of the interface layer) is in a perfect Svedrup balance with the wind. In this situation, the "Memory of the Ocean" is represented by the shape of the sea surface $\eta=\eta^{(eq)}$, which can be obtained from (1a) and (1b) using hydrostaticity (i.e. $v_{1\theta}=v_{1r}=0$). By doing so, we get, for the shape of sea surface, the following equation

$$\frac{\partial \eta^{(eq)}}{\partial r}=\frac{\tau_r}{g\cdot\rho_1\cdot(H_1+\eta^{(eq)}-h^{(eq)})} \tag{2a}$$

$$\frac{1}{r}\cdot\frac{\partial \eta^{(eq)}}{\partial \theta}=\frac{\tau_\theta}{g\cdot\rho_1\cdot(H_1+\eta^{(eq)}-h^{(eq)})} \tag{2b}$$

where, $h^{(eq)}$ is the interface layer at the hydrostatic equilibrium and we have used the definition $D_1=D_1^{(eq)} = H_1+\eta^{(eq)}-h^{(eq)}$. Note that, with this position, the effect of the wind is embedded in to the shape of the sea surface. Naturally, because deformations in the equilibrium free surface induce changes in the internal fluid pressure, also the equilibrium interface layer, through the deformation of the equilibrium free surface, is deformed by the action of the surface wind. The relationship between the shape of the sea surface and the shape of the interface layer at the equilibrium state can be obtained from (1c) and (1d) using hydrostaticity of the bottom layer (i.e. $v_{2\theta}=v_{2r}=0$), i.e.

$$\frac{\partial h^{(eq)}}{\partial r}=-\frac{g}{g'}\cdot\frac{\rho_1}{\rho_2}\cdot\frac{\partial \eta^{(eq)}}{\partial r} \tag{2c}$$

$$\frac{\partial h^{(eq)}}{\partial \theta}=-\frac{g}{g'}\cdot\frac{\rho_1}{\rho_2}\cdot\frac{\partial \eta^{(eq)}}{\partial \theta} \tag{2d}$$

where $g' = g\cdot(\rho_2-\rho_1)/\rho_1$ is the reduced gravity. Note that, at this point, the concept of sub-surface memory of the ocean is completely specified: the equilibrium interface layer, which parameterizes the deformation of isopycnal surfaces in a continuously stratified ocean, and the shape of the equilibrium sea surface contain the "memory" of the surface wind. In the following we will seek for linear solutions to equation (1) excited by "small perturbations" around the equilibrium state defined by $\eta=\eta^{(eq)}$ and $h=h^{(eq)}$. From the mathematical point of view this approach is convenient, because it incorporates the effect of the wind stress in to the shape of the interface and surface layer and, as we shall see, the resulting equations are independent of the wind stress. The wind stress determines the shape of the equilibrium free surface though equations (2a) and (2b), and the shape of the equilibrium interface is determined by the shape of the equilibrium free surface through equations (2c) and (2d), which are automatically satisfied by putting $h^{(eq)}=-(g/g')\cdot(\rho_1/\rho_2)\cdot\eta^{(eq)}$ or, which is the same, $\eta^{(eq)}- h^{(eq)}=(g/g')\cdot\eta^{(eq)}$. At this point we are ready to seek for liner solution to (1) excited by small perturbations (σ, p) of the surface and interface layer around the equilibrium state, i.e. $\eta = \eta^{(eq)}+\sigma$, $h = h^{(eq)}+p$, $D_1 = H_1 + (\eta^{(eq)}- h^{(eq)})+(\sigma-p) = D_1^{(eq)}+ (\sigma-p)$ and $D_2 = H_2 + h^{(eq)}+p = D_2^{(eq)}+p$, but some care has to be taken because of a feedback induced by the

perturbation over the acceleration due to the wind stress (last term on the rightmost side of eq.s (1a) and (1b)). Indeed, at the equilibrium state, the acceleration is given by equation (2a) and (2b), which we summarize, dropping the subscripts θ and r to simplify the notation, as $\tau/(\rho_1 D_1^{(eq)})$; at the perturbed state the acceleration due to the wind stress is $\tau/(g\cdot\rho_1 D_1)$, which is different from the acceleration due to the wind stress at the equilibrium state because of the term $D_1 = D_1^{(eq)} + (\sigma\text{-}p)$ at the denominator. In order to express the acceleration due to the wind stress at the perturbed state in terms of acceleration due to the wind stress at the equilibrium state, we observe that a Taylor expansion provides $\tau/(\rho_1 D_1) \approx [\tau/(\rho_1 D_1^{(eq)})]\cdot[1-(\sigma\text{-}p)/D_1^{(eq)} +\ldots]$. Substituting this expression into, for instance (1a) (similar considerations stand for equation (2b)), using equation (2a) and the definition $\eta = \eta^{(eq)}+\sigma$, one gets, at the rightmost side of eq. (1), $-g\partial\sigma/\partial r - [\tau/(\rho_1 D_1^{(eq)})]\cdot(\sigma\text{-}p)/D_1^{(eq)}$. The latter term, even if it might be important because it could be responsible for the asymmetry of the BiOS phases, is difficult to handle, because it couples the motion in the surface and bottom layer. However, differently from most of the regions in the world ocean, where the average depth of the active surface layer is *150-200 m*, in the Ionian, we have seen in the main text that the dynamically active surface layer is composed by waters of Atlantic and Levantine origin and its deph is, approximately, 500 m. Therefore, we may assume that the term $[\tau/(\rho_1 D_1^{(eq)})]\cdot(\sigma\text{-}p)/D_1^{(eq)}$ represents a "small correction" and set, in equations (1a) and (1b), $\tau_{r,\theta}/(\rho_1 D_1) \approx \tau_{r,\theta}/(\rho_1 D_1^{(eq)})$. With this approximation in mind, substituting equation (2a, b) into (1a, b), after linearization we get the following equation of motion

$$\frac{\partial v_{1r}}{\partial t} - f v_{1\theta} = -g\cdot\frac{\partial\sigma}{\partial r} \tag{3a}$$

$$\frac{\partial v_{1\theta}}{\partial t} + f v_{1r} = -g\cdot\frac{1}{r}\cdot\frac{\partial\sigma}{\partial\theta} \tag{3b}$$

$$\frac{\partial v_{2r}}{\partial t} - f v_{2\theta} = -g\cdot\frac{\rho_1}{\rho_2}\cdot\frac{\partial\sigma}{\partial r} - g'\frac{\partial p}{\partial r} \tag{3c}$$

$$\frac{\partial v_{2\theta}}{\partial t} + f v_{2r} = -g\cdot\frac{\rho_1}{\rho_2}\cdot\frac{1}{r}\cdot\frac{\partial\sigma}{\partial\theta} - g'\cdot\frac{1}{r}\cdot\frac{\partial p}{\partial\theta} \tag{3d}$$

$$\frac{\partial(\sigma-p)}{\partial t} + \frac{1}{r}\cdot\frac{\partial\left[r\cdot\left(H_1+\eta^{(eq)}-h^{(eq)}\right)\cdot v_{1r}\right]}{\partial r} + \frac{1}{r}\cdot\frac{\partial\left[\left(H_1+\eta^{(eq)}-h^{(eq)}\right)\cdot v_{1\theta}\right]}{\partial\theta} = 0 \tag{3e}$$

$$\frac{\partial p}{\partial t} + \frac{1}{r}\cdot\frac{\partial\left[r\cdot\left(H_2+h^{(eq)}\right)\cdot v_{2r}\right]}{\partial r} + \frac{1}{r}\cdot\frac{\partial\left[\left(H_2+h^{(eq)}\right)\cdot v_{2\theta}\right]}{\partial\theta} = 0 \tag{3f}$$

As it is common practice in Internal Kelvin Wave theory, we shall seek for solutions to (3) with radial velocity $v_{ir} = 0$ and $p = \mu\cdot\sigma$ with μ independent of time. With this position, differentiating equation (3e) with respect to time, we get

$$(1-\mu)\cdot\frac{\partial^2\sigma}{\partial t^2} + \frac{1}{r}\cdot\frac{\partial v_{1\theta}}{\partial t}\cdot\frac{\partial\left(H_1+\eta^{(eq)}-h^{(eq)}\right)}{\partial\theta} + \frac{1}{r}\cdot\left(H_1+\eta^{(eq)}-h^{(eq)}\right)\cdot\frac{\partial^2 v_{1\theta}}{\partial\theta\partial t} = 0$$

using (3b)

$$(1-\mu)\cdot\frac{\partial^2\sigma}{\partial t^2} - \frac{g\cdot\left(H_1+\eta^{(eq)}-h^{(eq)}\right)}{r^2}\cdot\frac{\partial^2\sigma}{\partial\theta^2} - \frac{g}{r^2}\cdot\frac{\partial\left(\eta^{(eq)}-h^{(eq)}\right)}{\partial\theta}\cdot\frac{\partial\sigma}{\partial\theta} = 0$$

or

$$(1-\mu)\cdot\frac{\partial^2\sigma}{\partial t^2} - \frac{g\cdot\left(H_1+\eta^{(eq)}-h^{(eq)}\right)}{r^2}\cdot\frac{\partial^2\sigma}{\partial\theta^2} - \frac{g}{g'}\cdot\frac{\tau_\theta}{r\cdot\rho_1\cdot\left[H_1+(g/g')\cdot\eta^{(eq)}\right]}\cdot\frac{\partial\sigma}{\partial\theta} = 0 \tag{4}$$

where the relationship $\eta^{(eq)} - h^{(eq)} = (g/g')\cdot\eta^{(eq)}$ has been used. In order to determine the proportionality factor μ between surface and interface displacements, one could differentiate eq. (3f) with respect to t to obtain, after eliminating $v_{2\theta}$ by using eq. (3d), an equation for σ and requiring this equation to be identical to eq. (4). By doing so we get an equation for μ

$$c^2=(H_2+h^{(eq)})\cdot(g\frac{\rho_1}{\rho_2}+g'\mu)\frac{1}{\mu}=\frac{g\cdot(H_1+\eta^{(eq)}-h^{(eq)})}{1-\mu} \tag{5}$$

which becomes

$$g'(H_2+h^{(eq)})\cdot(1-\mu)^2-g(H_1+H_2+\eta^{(eq)})(1-\mu)+g(H_1+\eta^{(eq)}-h^{(eq)})=0 \tag{6a}$$

Another popular way to rewrite (6a) consists in using the definition of internal wave velocity in equation (5). By doing so, eq. (6a) becomes an equation for the internal wave velocity, i.e.

$$c^4-g(H_1+H_2+\eta^{(eq)})c^2+(H_1+\eta^{(eq)}-h^{(eq)})(H_2+h^{(eq)})g\cdot g'=0 \tag{6b}$$

with solutions

$$c_\pm^2=\frac{1}{2}[g\cdot(H_1+H_2+\eta^{(eq)})\pm g\cdot(H_1+H_2+\eta^{(eq)})\cdot\sqrt{1-4\cdot(\frac{H_2+h^{(eq)}}{H_1+H_2+\eta^{(eq)}})\cdot(\frac{g'}{g})}]$$

In general, within the ocean $g'/g\ll 1$, so expanding the square root in the above equation we get

$$c_\pm^2\begin{cases}=g(H_1+H_2+\eta^{(eq)})[1-\frac{(H_1+\eta^{(eq)}-h^{(eq)})(H_2+h^{(eq)})}{(H_1+H_2+\eta^{(eq)})^2}(\frac{g'}{g})]=c_t^2\cdot[1-(\frac{c_c^2}{c_t^2})\cdot(\frac{g'}{g})]\\ =g'\frac{(H_1+\eta^{(eq)}-h^{(eq)})\cdot(H_2+h^{(eq)})}{H_1+H_2+\eta^{(eq)}}=c_c^2\end{cases} \tag{7}$$

where we have set

$$c_t^2=g(H_1+H_2+\eta^{(eq)})$$
$$c_c^2=g'\frac{(H_1+\eta^{(eq)}-h^{(eq)})\cdot(H_2+h^{(eq)})}{H_1+H_2+\eta^{(eq)}}$$

The first solution represents the barotropic part of motion while the second represents the baroclinic. Concentrating on the baroclinic part of motion, using (6), we have

$$\mu=1-(\frac{g}{g'})\cdot(\frac{H_1+H_2+\eta^{(eq)}}{H_2+h^{(eq)}})\approx-(\frac{g}{g'})\cdot(\frac{H_1+H_2+\eta^{(eq)}}{H_2+h^{(eq)}})\approx-\frac{g}{g'}$$

with these positions, equation (4) becomes

$$\frac{\partial^2\sigma}{\partial t^2}-\frac{c_c^2}{r^2}\cdot\frac{\partial^2\sigma}{\partial\theta^2}-\frac{1}{r}\frac{\tau_\theta(H_2+h^{(eq)})}{\rho_1\cdot(H_1+H_2+\eta^{(eq)})\cdot[H_1+(g/g')\cdot\eta^{(eq)}]}\cdot\frac{\partial\sigma}{\partial\theta}=0 \tag{8}$$

which describes damped oscillations around the equilibrium state.

2. Approximations, orders of magnitude and analytical solution to the Kelvin-like wave model

Equation (8) is a damped wave equation with velocity and damping depending on the radial and angular coordinate, difficult to handle analytically without specific approximations. For this reason, observe that a reasonable value for the deformation of the free surface between the center and the periphery of the eddy is 3 cm (Figure 3 of the main text); similarly, for the Ionian Sea, reasonable values are $\rho_1 \approx 1028.6\ kg/m^3$, $\rho_2 \approx 1029.2\ kg/m^3$, $H_1 \approx 500\ m$ and $H_2 \approx 1700\ m$, which provide $g' \approx 0.0057\ m/s^2$, $c \approx 1.49\ m/s$ and $(g/g')\cdot\eta^{(eq)} \approx h^{(eq)} \approx 51\ m$. To simplify eq. (8), therefore, we observe that

$$c_c = \sqrt{\frac{g'\cdot(H_1+\eta^{(eq)}-h^{(eq)})\cdot(H_2+h^{(eq)})}{H_1+H_2+\eta^{(eq)}}} \approx \sqrt{\frac{g'\cdot H_1\cdot H_2}{H}} \qquad (9a)$$

$$\gamma = \frac{\tau_\theta(H_2+h^{(eq)})}{\rho_1\cdot(H_1+H_2+\eta^{(eq)})\cdot[H_1+(g/g')\cdot\eta^{(eq)}]} \approx \frac{\tau_\theta H_2}{\rho_1\cdot H\cdot H_1} \qquad (9b)$$

where $H = H_1+H_2$.

At this point we seek for separate variable solutions to (8) with the approximations (9a) and (9b) of the form $\sigma(r,\theta,t)=P(t)\cdot\Sigma(\theta,r)$. Substituting this expression in to (8) and dividing by $P(t)\cdot\Sigma(\theta,r)$ we get

$$\frac{d^2P}{dt^2}+\alpha\cdot P=0 \qquad (10a)$$

$$\frac{c_c^2}{r^2}\cdot\frac{\partial^2\Sigma}{\partial\theta^2}+\frac{\gamma}{r}\cdot\frac{\partial\Sigma}{\partial\theta}+\alpha\cdot\Sigma=0 \qquad (10b)$$

Note that $[\gamma] = [L][T]^{-2}$, $[k] = [T]^{-2}$ and, naturally, $[c_c]=[L][T]^{-1}$. The general solution to (10b) is

$$\Sigma(\theta,r)=e^{-(\frac{\gamma}{2c_c^2})\cdot r\cdot\theta}[A\cdot\exp(ir\,\theta\cdot\frac{1}{2\cdot c_c}\cdot\sqrt{4\alpha-(\frac{\gamma}{c_c})^2})+B\cdot\exp(-ir\,\theta\cdot\frac{1}{2\cdot c_c}\cdot\sqrt{4\alpha-(\frac{\gamma}{c_c})^2})]$$

There are several ways to set the boundary conditions. Here we set $\Sigma(\theta,r)]_{\theta=0}=0=\Sigma(\theta,r)]_{\theta=\theta_0}$ with the condition that $r\cdot\theta_0/c_c$ *[= time necessary to the wave to travel the distance* $r\cdot\theta_0$*]* $>>2c_c/\gamma$ *[= time necessary to the wave to travel the damping distance* $2\,c_g^2/\gamma$ *]*. Using the first, we get B = -A and

$$\Sigma(\theta,r)=2\cdot i\cdot A\cdot e^{-(\frac{\gamma}{2c_c^2})\cdot r\cdot\theta}\cdot\sin(r\,\theta\cdot\frac{1}{2\cdot c_c}\cdot\sqrt{4\alpha-(\frac{\gamma}{c_c})^2})=2\cdot i\cdot A\cdot e^{-\frac{r}{L}\cdot\theta}\cdot\sin(k\cdot r\cdot\theta)$$

where we have set

$$k=\frac{1}{2\cdot c_c}\cdot\sqrt{4\alpha-(\frac{\gamma}{c_c})^2}$$

$$L=\frac{2c_c^2}{\gamma}$$

Using the second boundary condition we get the quantization condition on *k,* i.e.

$$k_m=\frac{m\pi}{r\theta_0}\Rightarrow\alpha_m=\omega_m^2=(\frac{\gamma}{2\cdot c_c})^2+m^2\cdot(\frac{c_c\pi}{r\cdot\theta_0})^2$$

with $m = 1....\infty$. Note that $[k_m] = [L]^{-1}$ and $[\omega_m] = [T]^{-1}$. With these positions, the general solution to (8) with the approximations (9a-b) is

$$\sigma(\theta,r,t)=\sum_{m=1}^{\infty} e^{-\left(\frac{r}{L}\right)\cdot\theta}\cdot\sin(k_m\cdot r\cdot\theta)\cdot[a_m(r)\cdot\cos(\omega_m t)+b_m(r)\cdot\sin(\omega_m t)]$$

Therefore, we may expect that for large values of θ_0 (i.e. $\theta_0 >> L/r$), the solution to (8) is composed by oscillations with frequencies close to $\approx (\gamma/2c_c)$. In our case r can be estimated as the radius of the Ionian eddy, that from Figure 3 of the main text is $\approx$ *80 km.*

3. Details on fitting the second Empirical Orthogonal Function temporal amplitude

Figure S2 shows the temporal amplitude of the second Empirical Orthogonal Function (EOF-black points). The red line represents the fitting function computed using the data between 1 Jan 1993 and 31 Dec 2016; the green line represents the fitting function computed using the data between 1 Jan 1996 and 31 Dec 2016. Nearly no change in the fitting function can be observed, indicating the Eastern Mediterranean Transient, which ended in 1995, has no role in determining the fitting function.

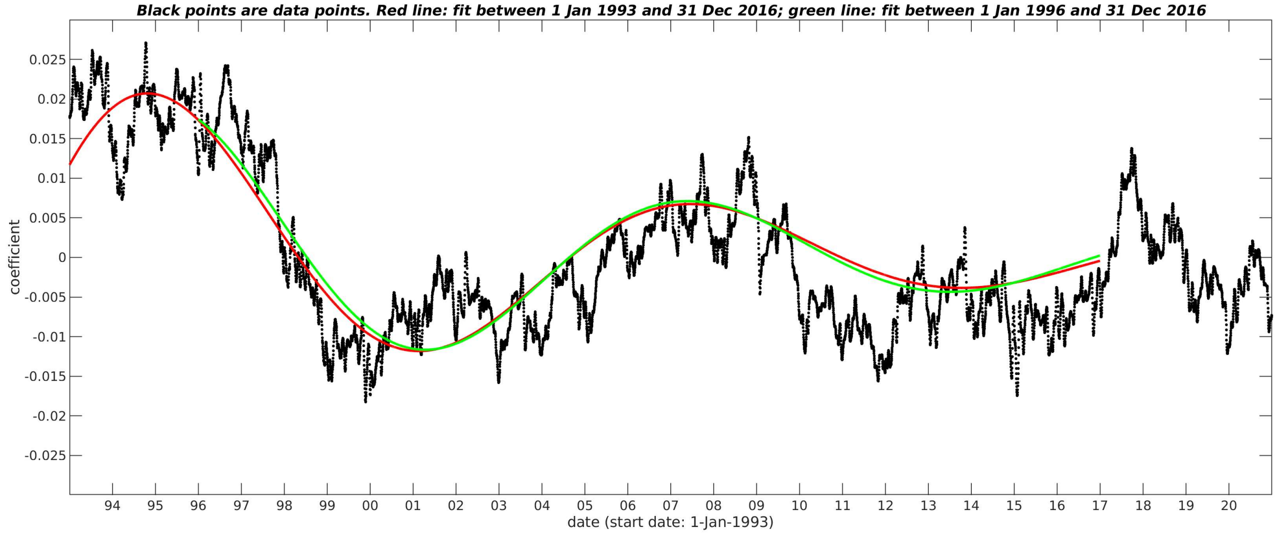

Figure S2. Multiple fitting of the second EOF temporal amplitude. Black point are data point. Red line: fitting between 1 Jan 1993 and 31 Dec 2016; green line: fitting between 1 Jan 1996 and 31 Dec 2016. In the second period the Eastern Mediterranean Transient was not active anymore. This figure has been created using MATLAB software package version 2018a (www.mathworks.com).

3. Details on Empirical Orthogonal Functions

Figure S3 shows the spatial mode 1, 3, 4 and 5 of the Empirical Orthogonal Function (EOF) analysis. Cumulatively, these modes along with the second EOF mode, explain 86.7% of the overall Sea Surface Height data set variance. Since no structure resembling the Northern Ionian Gyre can be detected in spatial modes 1, 3, 4 and 5, this implies that the second Empirical Orthogonal Function mode explains, at least, 86.7% of the Northern Ionian Gyre variability.

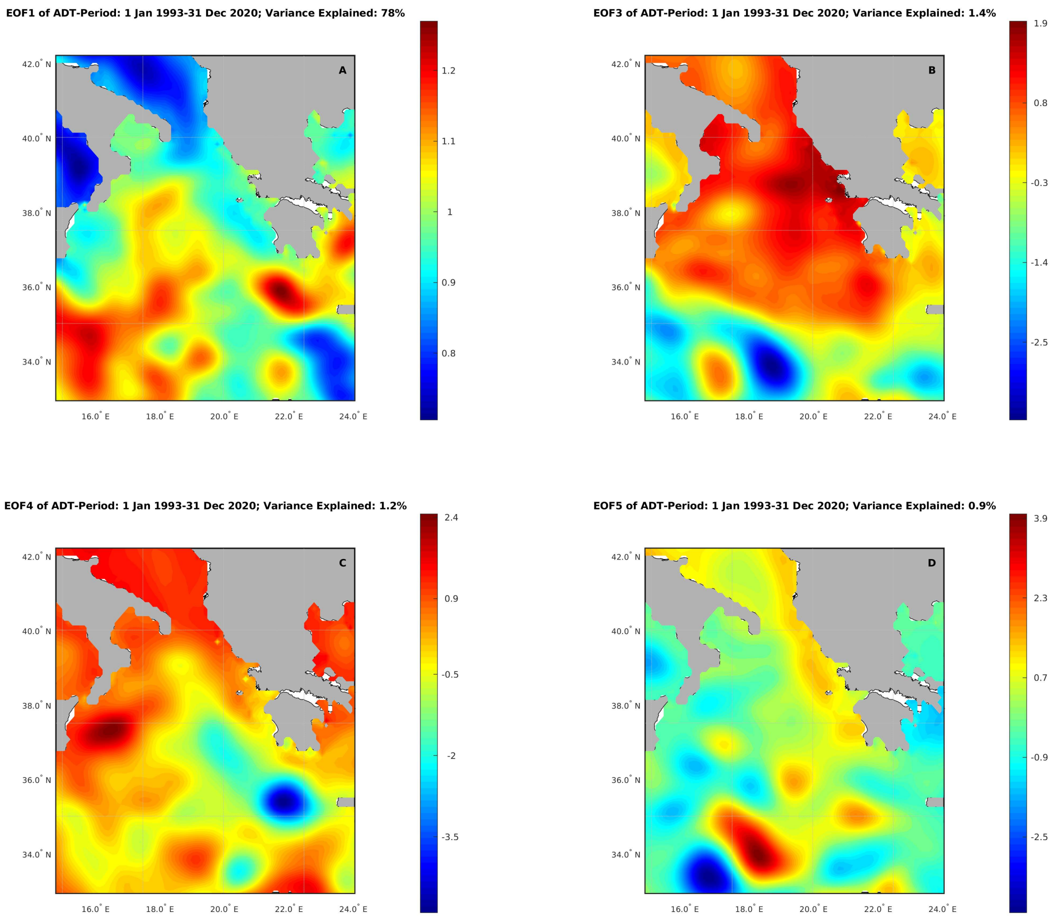

Figure S3. EOF 1, 3, 4 and 5 of Absolute Dynamic Topography over the study region. This figure has been created using MATLAB software package version 2018a (www.mathworks.com).

4. Details of the averaging regions quoted in the main body of the text

Throughout the text, spatial averaging procedures on “Study” and “Eddy” regions are mentioned. The following figure is meant to provide a synoptic view of these regions. Specifically, the “Study Region” (SR), which comprises, beside the Ionian, part of the Southern Adriatic, and a slight piece of the Cretan and Tyrrhenian sea is the entire region represented in Figure S4; the “Eddy Region” (ER) is the area depicted in the “Study Region”.

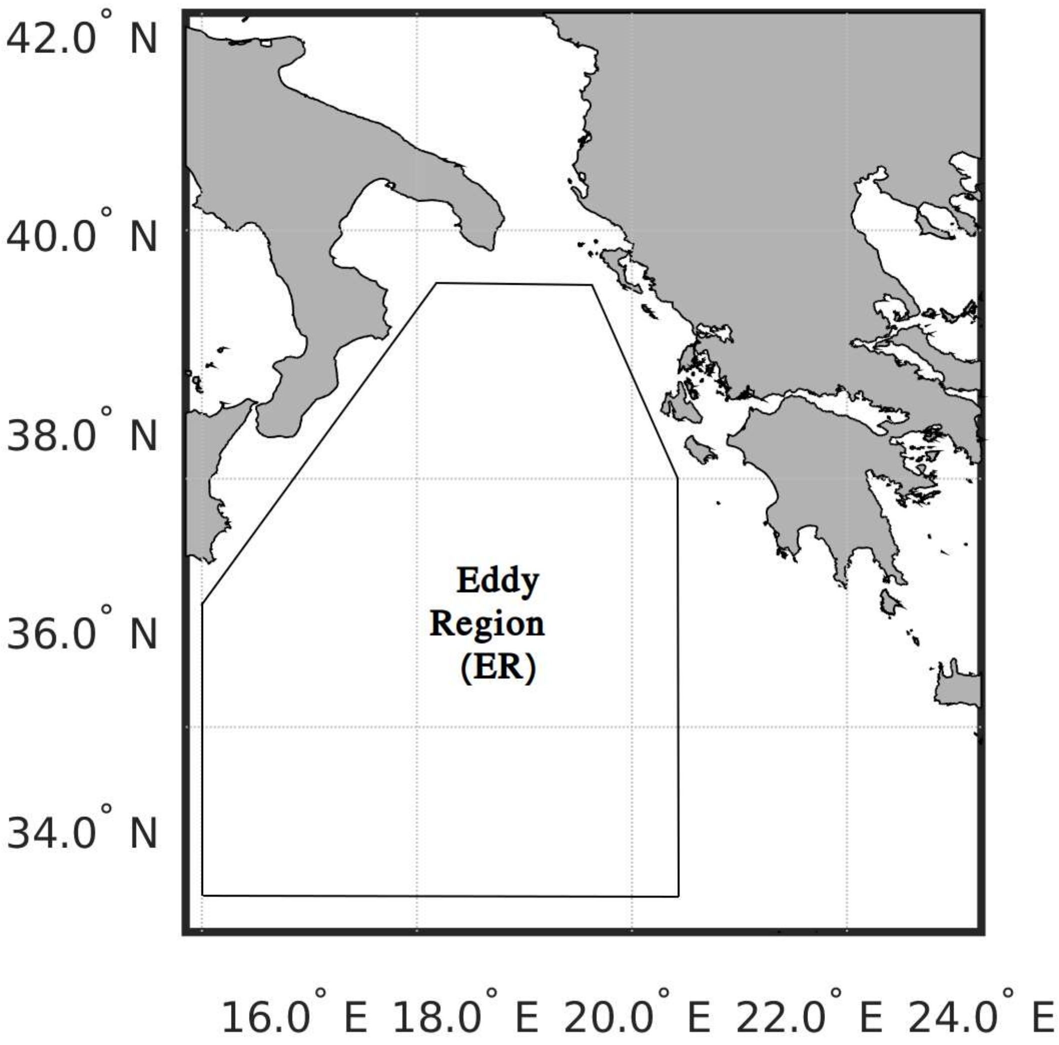

Figure S4. The overall area represents the “Study Region” (SR). The area depicted in the “Study Region” is the “Eddy Region” (ER). This figure has been created using MATLAB software package version 2018a (www.mathworks.com).
